# The impact of identified agility components on project success—ICT industry perspective

**DOI:** 10.1371/journal.pone.0281936

**Published:** 2023-03-23

**Authors:** Lukasz Kanski, Katarzyna Budzynska, Jan Chadam

**Affiliations:** Department of Information Systems and Logistics, Faculty of Economics, Institute of Management and Quality Sciences, Maria Curie-Sklodowska University, Lublin, Poland; Fayoum University Faculty of Computers and Information, EGYPT

## Abstract

**Context:**

The complexity of modern economic processes, along with the pressure from competition, the increasing client expectations and the need to introduce changes in the course of project implementation have led to a great interest in agile management methods. Therefore, the answer the question whether the implementation of an agile approach to project management can contribute to the project success is gaining relevance in a changing and inconsistent environment.

**Objective:**

This study attempts to answer the question which aspects of agility and in what manner can influence the final outcome of project work? The underlying objective of the study was to identify the conditions and success factors of implemented projects.

**Method:**

This paper presents the results of a survey conducted in companies in ICT sector. For this purpose, a number of reports and materials provided by organisations and associations dealing with project management issues, in particular ICT projects, were analysed, complemented with data obtained from statistical offices and financial institutions. For the purposes of the own study, an original questionnaire was developed to identify critical factors for project success, in particular the defined agile components.

**Results:**

On the basis of the conducted survey it can be concluded that the presence and high level agility is conducive to successful projects. It is the high level of agility supporting project activities that is essential for success. Of no less importance are the people and interactions between them, as well as self-organising teams. They are crucial for a positive evaluation of the project activities.

**Conclusions:**

Identification of the relationship between selected agility components and project success components allowed recommendations for solutions and attitudes that increase the chances of success in project management to be formulated. The synergy of individual agile components applied that increases the chances for project success.

## Introduction

The complexity of modern economic processes, along with the pressure from competition, the increasing client expectations are pushing forward the implementation of new approaches in managing the organisation and its activities. The development of enterprises is increasingly project-oriented and to a large extent is dependent on the success of implemented projects. The very issue of effective project management is the subject of numerous scientific and business debates. In the literature on the subject one can find many publications on project management and its various definitions. This is due to the fact that succeeding in project tasks invariably remains a major challenge for managers. The more complex the issue, the greater the uncertainty of the final results of the project. This is despite significant methodological progress and advancement in implementation that has been seen recently [1–6].

Choosing the right project management method may increase the success of a project. According to Pulse of Profession Report 2021 [7], 28% of agile method projects are successful, compared to 16% of projects managed in a traditional way. The analysis by Project Management Institute of key success factors has identified two main characteristics that can foster success with the use of agile management. These include high level of organisational flexibility and the implementation of standards in risk management practices [7].

The agile approach is still gaining momentum, however, most research on the importance of this group of methods compared to traditional methods is limited to conducting research on a small research sample, in the form of a case study [8–10] and a small number of elements influencing project success [11–14]. Projects in the ICT industry are diverse in nature, context, size and complexity [15], which is why this industry was chosen for the study described herein. The ICT (*Information and Communication Technologies*) sector is one of the industries that has seen the fastest growth in recent years. According to many authors, this sector has a major impact on economic growth [16–20], creation of new jobs, innovation in companies, lower labour costs or new economic opportunities [21]. The added value of the ICT sector in the EU in 2018 was EUR 479 billion, or 3.7% of EU GDP. Within the Member States group, the value added of ICT services between 2013 and 2018 increased by 26.8% and the value added of ICT production by 31% [22]. More in-depth research will provide information on the importance of agile methods for both researchers and practitioners to use in practice.

The validity of this problem is confirmed by the reviewed literature on the subject, which indicated a research gap in this area. Thus far, the research pertaining to agile approach and project success has focused on individual issues without attempting to identify their interdependence [23, 24]. Awareness of these dependencies opens a new perspective for management, focusing attention on the components of agile approach that support effective performance of undertakings. Consequently, the purpose of this paper was to identify the factors determining project success from the perspective of key components of agile approach.

Nowadays, companies decide to adopt a project-based approach more and more frequently, therefore identifying the relationships between various success factors is of crucial importance. Effective project implementation contributes to gaining competitive edge. Still, successful completion of project tasks remains a significant challenge for managers. The more complex components the project involves, the greater the uncertainty of the final outcome. This happens despite considerable progress in methodology and application of project-based approach [1, 4, 6, 25, 26]. However, it should be noted that the relations between project success and different success factors evolve depending on the maturity of an organisation. What is more, the factors may be perceived differently by individual project participants [5]. Most importantly, such an approach strengthens organisations in terms of knowledge sharing, and is conducive to the development of new, often revolutionary solutions, as well as supporting the company’s innovation capabilities [27–29].

As mentioned earlier, the complexity of processes in the modern world has forced companies to look for new solutions in project management. Empirical research conducted in the process of identifying the elements of agile methods that influence project success has been developed for 20 years and contributes important knowledge in this area. However, most studies do not answer the question which elements are widely used in agile methods and how they influence the final outcome of project work. Therefore, an attempt was made to answer these questions and with this in mind, the following research hypotheses were adopted:

1. **Choosing the right configuration of agile components is a fundamental key to success in project implementation**.
2. **Enterprise-recorded levels of individual agility components are similar to each other**.
3. **The levels of occurrence of project success components reported by enterprises are similar to each other**.
4. **The level of implementation of agile project management solutions in an enterprise affects eventual project success (a relationship exists between these components)**.
5. **Interdependencies exist between agile components and project success components**.

In the authors’ opinion, the completed research will extend our knowledge by providing information and analysis based on empirical quantitative research. The research covered but was not limited to the following issues:

1. Identification of the key elements of project success and the most important components of agility.
2. Determination of agility components that are commonly used in agile methods and how they influence the final outcome of project work.
3. Empirical evaluation of the relationship between project success and agile components.
4. Development of a set of application recommendations for practitioners based on the obtained research results.

The paper is divided into sections. The “Theoretical basis” section presents a literature review related to project success and agile methods. The “Research methodology” section describes the research method used. The survey results are presented in”The influence of agility factors on project success-findings” section and discussed in the following section. The limitations of the study and the possibility of further research are discussed in the “Limitations and future research” section. A summary of the results and their practical implications is included in the “Conclusions” section.

## Theoretical basis

### Project and project success—A definitional approach

There are many various definitions of the term "project” in the literature on the subject [e.g. [30–33]]. In the case of definitions provided by associations, institutes and organisations such as IPMA, Axelos or PMI, the emphasis is on the uniqueness of the process leading to the product. However, in the case of definitions provided by individual researchers, the uniqueness and specificity of the product is emphasised, although a clear connection of activities (production process) with the product is also noticed [34–39].

This paper assumes that the project is *a time-limited*, *performed in a fixed cost regime and within specified resources*, *unique*, *individual undertaking (organisation)*, *aimed at developing a complete*, *unique (in terms of features or process in which the development occurs) product or service*. The fundamental expectation related with project implementation is success. The attempt to properly define project success proves to be difficult in practice. This is due to the lack of an unambiguous criterion for evaluating the final result, in other words: the final result depends on the assumed assessment criterion.

The effective management in any project, regardless of its size, complexity, scope, definitional approach, etc., is driven by project success. The current knowledge—both theoretical and practical—in this area is very extensive, nonetheless, achieving the assumed objective(s) in project tasks invariably remains a major challenge for managers. The more complex the issue, the greater the uncertainty of the final results of the project, especially taking into account the risk and reliability of operation after commissioning (e.g. complex scope of work, dependence on subcontractors, compliance with regulations, diversity of the team, return on investment in the long term, etc.) [40, 41].

Due to the importance of project management to the organisation, the three traditional parameters of the "golden project triangle" defined in the 1970s by PMI (i.e. budget, schedule and functionality) are still relevant in today’s business environment [42, 43], however, they seem to be insufficient nowadays [44, 45]. On account of the ambiguity of project success definition, in addition to the above-mentioned list of "golden triangle" criteria, the terms "positive square” criteria or "quadruple constraint” are becoming more and more popular and additionally include the satisfaction of the client (investor) or the user of the project’s product. Therefore, a new approach has emerged to adapt to the new business environment and to make the existing project process more flexible [46–48]. In a study, Pinto and Slevin [49] presented a list of critical factors for project success that is most often cited in the literature [50]. From another perspective, there is a link between project success and the efficiency and effectiveness of project implementation [51–53]. In contrast to the search for a general measure of success, the literature also includes the *project contingency theory* (PCT) [54, 55]. Research based on the PCT theory describes four foundations for evaluating project success (NTCP): novelty, technology, complexity and pace.

In the course of extensive analysis of the effectiveness of project activities, eight key parameters for project success evaluation were identified and further used in this study. These include:

- keeping up with budget;
- keeping up with schedule;
- ensuring functionality;
- client satisfaction;
- satisfaction of project team members;
- ensuring benefits for recipients of project’s products;
- ensuring technical, organisational, social, political and business benefits;
- achieving the strategic objectives of the company.

In the following part of the paper, an attempt has been made to examine the influence of individual agile components on the components of project success and, consequently, to determine the impact of an agile approach on the successful implementation of a project task. The analysis has been carried out using dedicated statistical methods.

### Theoretical foundations of agile methods

The systematic growth of project-based tasks has resulted in the dynamic development of project management methods in the context of the development and dissemination of operational research methods. Particularly significant changes in the approach to project management originate from the Manifesto for Agile Software Development (*Agile Manifesto*), which was published in 2001. Its consequence was a dynamic development of agile methods, including Scrum, Extreme Programming, Lean Management, and Dynamic System Development Method. The common feature of this group of methods is the implementation of the project in small fragments, in an iterative and incremental model, with the constant involvement of the client. Their natural feature is the lack of possibility of precise planning of the entire project, which in turn is related mainly to uncertainty in determining ways of achieving project objectives. Therefore, these methods have found many supporters especially in projects of an innovative nature, with a defined objective but unknown or uncertain ways of achieving it [56–60]. However, it is worth noting that projects implemented using agile methods are more and more common, where both the objective and the ways of achieving it are undefined, and the research covers both successful and unsuccessful implementation of the project [11, 61, 62]. Agile methods are most often used in companies that are organisationally mature and open to new challenges. They offer a chance to quickly achieve project results, assuming that an optimal way of performing project tasks has been found.

The literature provides carious definitions of agility [e.g.[63–67]]. For this study, a comprehensive definition of agility developed by Conboy, has been adopted. In the said definition, its author substitutes the ability of an entity for the continual readiness of an entity to: “the continual readiness of an ISD method to rapidly or inherently create change or to proactively or reactively embrace change through its internal components and relationships with its environment” [68].

The rise of agile methods’ popularity is becoming a global trend in project management, especially in ICT projects. Due to the specifics of the implemented projects, the approach presented in these methods is increasingly preferred by both clients and solution providers in various industries. However, the observation of the market leaves no doubt that when implementing agile project management methods, a number of difficulties, limitations and barriers may be encountered [7, 69]. The main differences between traditional and agile project management approaches can be seen in the requirements and specifications, project schedule, collaboration within the team and with a client [70]. Naturally, an agile approach to project management does not completely reject following any plans, but it fosters responding to changes during the performance of the work more than strictly following a schedule. Likewise, agility does not reject processes and tools, complete documentation or official arrangements in the form of agreements, annexes or contracts, but values human interaction, software that meets client requirements, and the quality of collaboration with the client. A common feature of agile methods is the implementation of the project in small fragments, in an iterative and incremental model, where at the end of each iteration the client receives working software representing a measurable business benefit. The principles of cooperation of the parties involved, such as organisational independence of the team, co-responsibility of the team for the final effect of the project, self-motivation and self-sufficiency of the team, or trusting in team members have been described in many scientific works [e.g. [70–75]].

For the purpose of assessing and distinguishing the different components of agility, a detailed analysis of the *Agile Manifesto* has been carried out, based on its underlying principles [76]. These principles are used in each of the selected agile approaches, although they may be understood, interpreted or implemented differently in everyday development practice. However, in the course of a thorough analysis of both the *Agile Manifesto* itself, as well as the underlying principles of a number of agile methods, nine common agile components have been identified that are pivotal to this approach, regardless of the method adopted. These include:

- people and interactions over tools and processes;
- working software prevailing over detailed documentation;
- client collaboration prevailing over contract negotiation;
- responding to changes in the course of work prevailing over following a plan;
- delivering project deliverables in an iterative, incremental manner;
- the best architecture, requirements and design solutions originating from self-organising teams;
- maintaining good relationships with project stakeholders, characterised by mutual trust and cooperation;
- performance and functional criteria used when evaluating offers (samples, system demonstrations);
- project meetings (sprints) organised frequently enough.
- The elements listed above were subject to further analysis.

## Research methodology

### Research model, objectives, hypothesis

**Fig 1:**
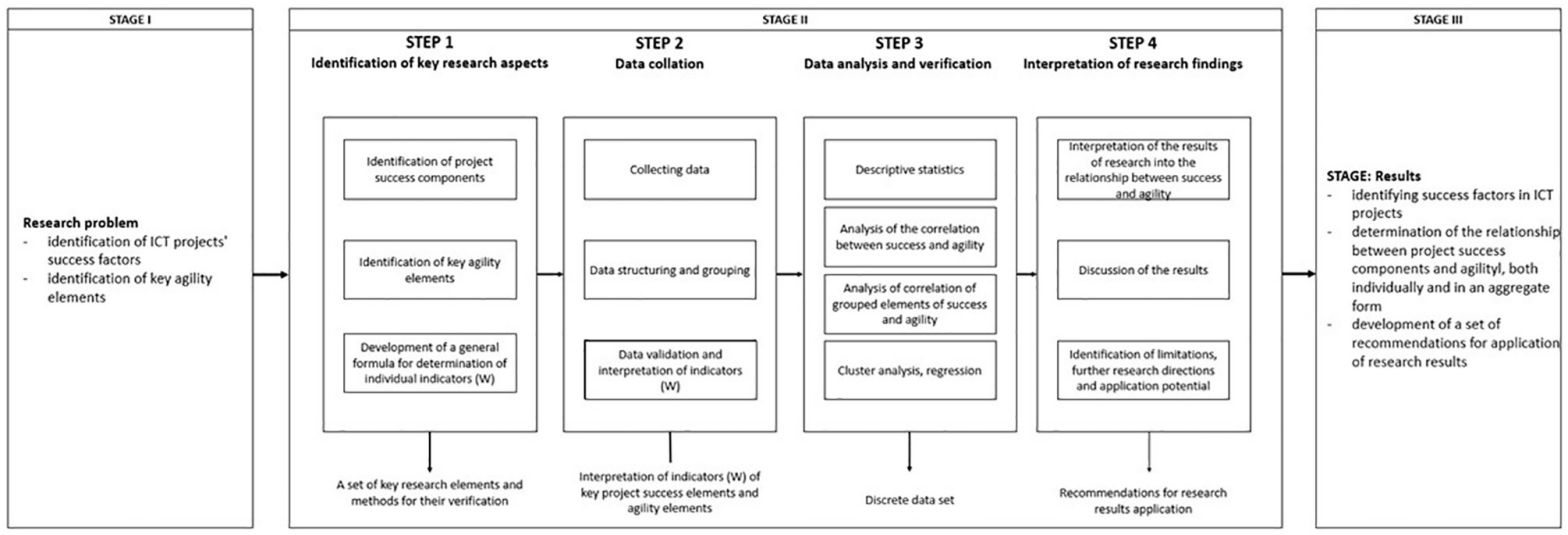
Research model scheme. Source: own study.

The adopted research model (Fig 1) included the stage of defining the research problem and formulating the research objective (Stage I), four intermediate steps related to the implementation of the survey (Stage II) and a stage of discussing the results with the development of recommendations for application (Stage III).

In stage two: in step one the key elements of project success and the most important components of agility were identified. Step two involved the process of collecting, aggregating and validating the collated data. Also at this point the results obtained were interpreted, in particular regarding the assessment of project success elements and agility. Step three was the key stage in the study. The research sample was characterised and the statistical methods applied for data analysis were aimed at verifying the relationship between project success and agility components. The last (fourth) step involved the interpretation of survey results, in particular the determination of the relationship of agility components and project success components, as well as project success in aggregated form using the developed formula for determining indicators (*W*). The result of this step was the development of a set of practical recommendations.

The main reason for undertaking the study was an attempt to answer the question which agility components are commonly used in agile methods and how they influence the final outcome of project work. The analysis of the problems outlined in this way led to the definition of the research problem as follows: there are competence gaps on the occurrence of relations between project success and agile components. The underlying objective of the study was to identify the conditions and success factors of implemented projects, especially ICT ones, in the context of commonly applied agile methods.

### Research method

The application of the adopted research methods was preceded by a review of literature on the conditions and factors conducive to project success and issues concerning organisational culture. Additionally, the authors conducted an extensive review of reports and materials made available by organisations and associations dealing with project management issues, in particular ICT projects [see e.g: [30, 77]] and data provided by statistical offices and financial institutions. For the purposes of the own study, a questionnaire was developed to determine critical project success factors, in particular the identified agility components.

The author’s questionnaire was the primary research tool. It contained five sections and a total of 88 questions exploring 155 features. The responses to most questions were based on five-point Likert scale This scale is a bipolar interval scale that measures respondent’s attitude and beliefs. Bipolarity implies two opposite poles at both ends of the scale reflecting opposing beliefs. Interval scale indicates that consecutive points of the scale are ordered and the distance between them is equal. The Likert scale was interpreted in a traditional way (1—strongly disagree, 2—rather disagree, 3—neutral position, 4—rather agree, 5—strongly agree). In part of the survey questions, dedicated measurement scales were used depending on the purpose of the survey and the adopted research method (nominal, dichotomous, ordinal and interval scales).

### Statistical data analysis methodology

The data collected in the survey were analysed in two phases. Firstly, the source data were analysed (without transforming). In the second phase, on the basis of collected data, several indicators were developed (transformed source data) and used for further analyses.

The collected data were analysed using selected elements of descriptive statistics (frequency analysis, analyses using mean, median, mode, skewness, kurtosis, standard deviation), cross-tabulations (dashboards) and statistical tests (chi-square test, Yates continuity correction, Cramer V coefficient, coefficient of determination, reliability quotient, linear relationship test). The analysis of reliability of the adopted scales was performed using Cronbach alpha coefficient as a technique of measuring their homogeneity.

For the groups of questions selected in the survey questionnaire the author’s formula for determining the value of indicators (*W*) of fulfilment of the set criteria was developed (formula 1). These indicators were used to establish the degree of implementation of selected aspects of project success and to assess individual elements of agility, and consequently to determine the level of agility and success of the entire project undertaking. Categorical variables that took values from 1 (strongly disagree) to 5 (strongly agree) were divided into three categories: 0 (strongly disagree, disagree), u (unconclusive), 1 (agree, strongly agree).

Formula 1. General formula for the determination of the indicator (*W*)

$$W=\left\{\begin{array}{c} 0,\;\;for\;\Sigma_{i=1}^{n}\;x_{i}<3n\\ unconclusive,\;for\;\Sigma_{i=1}^{n}\;x_{i}=3n\\ 1,\;for\;\Sigma_{i=1}^{n}x_{i}>3n \end{array}\right.$$ (1)

*W*—designated indicator,

*n*—number of elements (taken into account in creating the indicator),

*i*—consecutive number of the analysed response (assessment),

*x*_*i*_- value of the response (assessment), expressed by the respondent for the n^th^ object included in the indicator, *x*_*i*_ ∈ {1,2,3,4,5}.

The resulting *W* index was interpreted as follows:

- 0—means the value of the indicator for the sum of points smaller than 3*n*—was interpreted as condition unfulfilled,
- *Inconclusive*—means that a value of 0 or 1 cannot be assigned to an indicator for a sum of points equal to 3*n*,
- 1—means the value of the indicator for the sum of points above 3*n*, interpreted as fulfilling the condition.

As a limit of the positive verification of each determined indicator a middle value was adopted (3*n*) between the minimum (*n*) and maximum (5*n*) value of obtained points for each determined indicator was assumed. The minimum value of possible points determining a given indicator (feature) is a product of the number of responses and the value of 1 (from a five-point Likert scale). The maximum value for a given indicator (feature) is the product of the number of responses and the value of 5 (from the five-point Likert scale).

With the use of processed data, an attempt was made at developing a model (analysis of multiple and logistic regression) of the project success’s dependency on identified components of agile approach. The research and statistical methods adopted, models and indicators applied and their interpretations are recommended for implementation in management science and qualitology [78, 79].

### Characteristics of survey data

The adopted research method consisted in the development of a questionnaire, analysis and interpretation of survey results, and discussion of research implications. Survey respondents were selected based on purposive sampling. The participants were required to have the experience in managing large-scale investment projects, as well as in development of corporate standards on project management practice (representatives of company management, product managers, project managers and users). The survey covered persons participating in project implementation in the ICT area in the broad sense, and providing advisory and consulting services in this field, in particular external experts and contract engineers. The scope of the research included an analysis of the role of the ordering party/investor and the contractor/supplier in the project. On the part of the contractor, respondents included project managers, product managers and company executives. On the part of the investor, project managers and management representatives formed the group of respondents. With the above assumptions in mind, the survey questionnaire was addressed to employees in the ICT industry, mainly public companies. The survey was conducted in enterprises with their registered office or branches in Poland, but included projects implemented on the international, mainly European market. The basic criterion for qualifying a company to participate in the survey was its entry in the major industry report in Poland, i.e. ’*Computerworld Top 200*’ 2021. The report ranks the leaders of the Polish ICT market, both consumers and suppliers of IT solutions and services in various sectors, including: industry, manufacturing, construction, agriculture, processing, public administration, finance, insurance and others. The questionnaire was addressed to all 367 companies included in the report. Respondents’ participation in the survey was voluntary. A total of 288 complete questionnaires were returned. The survey was carried out with the use of LimeSurvey online tool, while statistical analyses were performed with the use of IBM SPSS Statistics and StatSoft Statistica software. Respondents completed the survey in an electronic format, following the instructions provided on the website.

The proper survey was preceded by a pilot study on a group of 33 respondents and direct interviews with selected respondents (in-depth interview, IDI). The sample was purposeful and included experienced and professional project managers. Such approach was adopted with a view to selecting individuals who consciously and reliably evaluated the analyses conducted and the questionnaire described above. The purpose of those actions was to verify the questionnaire adequacy in terms of its proper understanding by the respondents, transparency and the suitability of the adopted research methods. The information gathered during the pilot survey and face-to-face interviews was used to develop the final version of the questionnaire which was used to conduct the proper survey.

In the cases analysed, 15% of the respondents represented the role of the ordering party, whereas the role of the contractor was represented by 85% of the respondents. The vast majority of respondents represented medium-sized enterprises, accounting for 70%. It should be noted that 84% of survey respondents were employed in enterprises operating on the market for over 5 years. Over 54% of respondents indicated IT as their leading industry, and over 23% indicated the telecommunications industry. This means that nearly 80% of the respondents participating in the survey work in the ICT industry. 68% of respondents were between 36 and 50 years old. The most numerous group of respondents constituted project managers—31%. 70% of survey participants were experienced employees with over 6 years of professional background, of whom 21% had over 10 years of experience in project management. The analysis of survey results indicated that 55% of respondents participated in projects involving between 11 and 50 people. Participation in projects carried out by more than 50 people was declared by 26% of respondents. A brief summary of the survey sample characteristics is presented in Table 1.

**Table 1:** Characteristics of the survey sample.

**Characteristics of enterprises represented by survey respondents**	
Role of enterprises in project implementation	
Contractor	85%
Employer	15%
Categories of surveyed enterprises by size	
Small	17%
Medium	70%
Large	13%
Period of operation of the company on the market	
Up to 1 year	2%
Between 1 and 5 years	14%
Between 6 and 10 years	62%
Over 10 years	22%
Discipline	
IT	54%
Telecommunications	23%
Energy	10.7%
Industry	4%
Financial services	2%
Marketing and advertising	2%
Construction	0.3%
Other (medical, military)	4%
**Characteristics of respondents participating in the survey**	
Age of respondents	
Up to 25 years	4%
Between 26 and 35 years	24%
Between 36 and 50 years	68%
Over 50 years	4%
Characteristics of respondents’ jobs	
Product user	4%
Project team member	12%
Project manager	31%
Lower/middle level manager	18%
High-level manager	28%
President/Member of the Board	7%
Respondents’ professional experience	
Less than 1 year	6%
Between 2 and 5 years	24%
Between 6 and 10 years	49%
Over 10 years	21%
Territorial area of the implemented projects	
Nation-wide projects	30%
International projects	70%
Number of people involved in the implementation of a single project	
From 1 to 10 persons	19%
From 11 to 50 persons	55%
From 51 to 100 persons	22%
More than 100 people	4%
Complexity of the projects	
Simple projects	4%
Moderately complex projects	21%
Complex projects	62%
Highly complex projects	13%

Source: own study. N = 288.

## The influence of agility factors on project success—Findings

### Analysis of the extent of involvement of individual agile components

The first part of analysis was focused on the extent of involvement of individual agility components that play a key role in the implementation and use of agile methods. The respondents were asked 9 questions to determine the degree of correspondence of the individual agility components. The value of Cronbach alpha coefficient (*AC* = 0,928) indicates the correct choice of the set of variables adopted to determine the type of project management method used. The results of data analysis of the individual project success parameters are summarised in Table 2.

**Table 2:** Identification of the extent of involvement of individual agility components in project task implementation.

Agility components	Mean $\bar{X}$	Median *M*(*X*)	Mode *D*(*X*)	Standard deviation *S*(*X*)	Skewness *A*(*X*)	Kurtosis *K*(*X*)
People and interactions prevailing over tools and processes	3,14	4,00	4,00	1,575	-0,188	-1,604
Working software prevailing over detailed documentation	3,20	4,00	4,00	1,561	-0,238	-1,569
Client collaboration prevailing over contract negotiation	2,88	4,00	4,00	1,549	-0,018	-1,635
Responding to changes in the course of work prevailing over following a plan	3,30	2,00	5,00	1,535	-0,267	-1,543
Delivering project deliverables in an iterative, incremental manner	3,07	4,00	4,00	1,525	-0,213	-1,564
The best architecture, requirements and design solutions originating from self-organising teams	3,27	4,00	5,00	1,590	-0,232	-1,614
Maintaining good relationships with project stakeholders, characterised by mutual trust and cooperation	3,07	4,00	4,00	1,539	-0,187	-1,579
Performance and functional criteria used when evaluating offers (samples, system demonstrations)	3,20	4,00	4,00	1,531	-0,190	-1,566
Project meetings (sprints) organised frequently enough	3,07	4,00	4,00	1,525	0,189	-1,566

Source: own study. N = 288.

Based on the above indicators, it can be concluded that in vast majority the respondents indicated the presence of agile components in the implemented works, which implies that agile methods are used in project management ($\bar{X}>3,0$). In addition, respondents indicated responding to changes during the implementation of works prevailing over following the plan as a key component of agility (top mean) ($\bar{X}=3,30$). This result is interesting due to the fact that the aforementioned agile component is a basic assumption of agile project management methods. The lowest average value ($\bar{X}=2,88$) was obtained for cooperation with the client over contract negotiation, which confirms the lack of trust and lack of full openness to an agile project management approach. The mean for the remaining components ranged from 3.07 to 3.20, which, although it indicates that individual agile components are present in the projects, is not the result that can be considered satisfactory.

The analysis as well as the values of the other indicators presented in Table 2 **positively verified H2, which means that the levels of the individual agility components recorded in companies are similar to each other**.

Based on agility components, indicators of achieving a certain level of individual agility components were additionally determined according to Formula 1. Using these indicators, it was determined whether according to respondents a certain level of each agility component was achieved (indicators were estimated for each case in the research sample). Next, the frequencies of three distinguished groups were counted (negative verification, not resolved, positive verification—Table 3). In most of the projects evaluated by the respondents, the indicator of partial agility components was more often verified positively than negatively.

**Table 3:** Assessment of agility components (%).

Project success components	Negative verification of the indicator	Inconclusive	Positive verification of the indicator
People and interactions prevailing over tools and processes	44%	0%	56%
Working software prevailing over detailed documentation	43%	0%	57%
Client collaboration prevailing over contract negotiation	51%	0%	49%
Responding to changes in the course of work prevailing over following a plan	42%	0%	58%
Delivering project deliverables in an iterative, incremental manner	43%	0%	57%
The best architecture, requirements and design solutions originating from self-organising teams	43%	0%	57%
Maintaining good relationships with project stakeholders, characterised by mutual trust and cooperation	44%	0%	56%
Performance and functional criteria used when evaluating offers	44%	0%	56%
Project meetings (sprints) organised frequently enough	42%	0%	58%

Source: own study. N = 288.

Further, the fact of using an agile approach was verified (aggregated form). The analyses were conducted with the use of the answers to all questions (*n* = 9) concerning the assessment of the elements forming the agility indicator—according to formula 1. The limit value indicating project success or failure was 27. For the criterion thus adopted, the frequency of agility occurrence or its absence was determined. The analysis shows that 56% of respondents indicated more frequent use of agile approach than traditional (waterfall) in the implemented projects. The opposite situation was indicated by 44% of the respondents. This result is considered satisfactory, as the participation of people using agile approach in the survey enabled the investigation of actual conditions of project success in the context of implementing agile methods project management.

### Analysis of project success

In the following step (in an analogous manner), the factors perceived as key components of project success were analysed. The selection of such elements was based on an extensive review of literature on the subject, as described in the first part of the paper. The respondents were asked 8 questions determining the degree to which the individual components of project success were met (5-point Likert scale). The value of Cronbach alpha coefficient (*AC* = 0,931) indicates that the set of variables adopted to determine the success of the project was properly selected. The results of data analysis of the individual project success parameters are summarised in Table 4.

**Table 4:** Test results for individual components of project success.

Project success components	Mean $\bar{X}$	Median *M*(*X*)	Mode *D*(*X*)	Standard deviation *S*(*X*)	Skewness *A*(*X*)	Kurtosis *K*(*X*)
Keeping up with budget	3,56	4,00	4,00	1,413	-0,647	-1,047
Keeping up with schedule	3,43	4,00	4,00	1,398	-0,553	-1,143
Ensuring functionality	3,32	4,00	4,00	1,400	-0,493	-1,224
Client’s satisfaction	3,58	4,00	5,00	1,489	-0,669	-1,107
Satisfaction of project teams’ members	3,35	4,00	4,00	1,484	-0,477	-1,313
Ensuring benefits for the recipients of the project products	3,50	4,00	4,00	1,374	-0,628	-1,021
Ensuring technical, organisational, social, political and business benefits	3,53	4,00	4,00	1,448	-0,656	-1,068
Achieving the strategic objectives of the company	3,46	4,00	5,00	1,502	-0,416	-1,427

Source: own study. N = 288.

The mean values ($\bar{\text{X}}$) in the eight analysed variables ranged from 3.32 to 3.58. The highest values were obtained for the areas of client satisfaction ($\bar{X}=3,58$) and keeping up with budget ($\bar{X}=3,56$). It means that ensuring benefits for clients and compliance with financial assumptions are particularly relevant. The results suggest that in most cases analysed, the project implementation summary and meeting the assumed objectives indicate project success, with the reservation that these do not necessarily mean full achievement of all the assumed project objectives.

The performed analysis and the values of indicators presented in Table 4 **confirm H3, which means that the levels of occurrence of project success components recorded by enterprises are similar to each other**.

Based on project success components, additional indicators were developed for the achievement of a certain level by individual components of project success—according to formula 1. On the basis of the above indicators it was determined whether, according to the respondents, a certain level of particular components of project success was achieved (indicators were estimated for each case in the surveyed sample). Next, the frequencies of three distinguished groups were counted (negative verification, not resolved, positive verification—Table 5). In the projects assessed by the respondents, partial success components indicator was verified positively more often than negatively.

**Table 5:** Ratings of project success components (%).

Project success components	Negative verification of the indicator	Inconclusive	Positive verification of the indicator
Keeping up with budget	32%	0%	68%
Keeping up with schedule	34%	0%	66%
Ensuring functionality	36%	0%	64%
Client’s satisfaction	32%	0%	68%
Satisfaction of project teams’ members	36%	0.5%	63.5%
Ensuring benefits for the recipients of the project outputs	32%	0%	68%
Ensuring technical, organisational, social, political and business benefits	35%	0%	65%
Achieving the strategic objectives of the company	32%	0%	68%

Source: own study. N = 288.

Further, the fact of project success was verified (aggregated form). The analyses were conducted using the answers to all questions (*n* = 8) concerning the evaluation of elements forming the project success indicator—according to formula 1. The limit value indicating project success or failure was 24. For this criterion, the frequency of project success and failure was determined. The results of the analysis demonstrate that 67% of respondents indicated that the undertakings more frequently turned out to be a success than a failure. The opposite situation was indicated by 33% of the respondents. This result can be considered satisfactory, as the participation of persons with track record of successful projects allowed for the examination of actual prerequisites for project success.

### Agility and project success

The analyses described so far focused on agility components and project success factors separately. These two areas are juxtaposed below. Current research on the effectiveness of agile methods leaves no doubt that their implementation is a prerequisite for project success. Identical conclusions emerge from the analysis of the own study described below. First of all, for the purposes of the analysis, the answers concerning success and failure were compared with the information on the type of project management method applied. Table 6 shows the contingency of the influence of the applied project management method on the final result of the project.

**Table 6:** Impact of the adopted project management method on project result.

		Project result		TOTAL
		Failure	Success	
Project management method	None	6%	4%	10%
	Standard	18%	15%	33%
	Agile	8%	48%	57%
SUM		33%	67%	100%

Source: own study. N = 288.

The analysis shows that when traditional methods are used, the evaluation of work implementation more often suggests failure (18%) than success (15%). The performance of work using agile methods resulted in project success in 48% of cases, whereby failure was reported in 8% of cases. In the cases where no standardised methods were applied, the result was comparable. The value of the chi-square test (χ^2^ = 53,036; *df* = 2; *p* < 0,001) indicates that a relationship exists between project implementation using established standards and recognised methods and project success. The φ value (*λ*(*x*) = 53,273; *df* = 2; *p* < 0,001, *φ* = 0,425; *p* < 0,001) and Cramer’s V statistic (*V Cramera* = 0,425; *p* < 0,001) however, are evidence of a moderate correlation.

**Fig 2:**
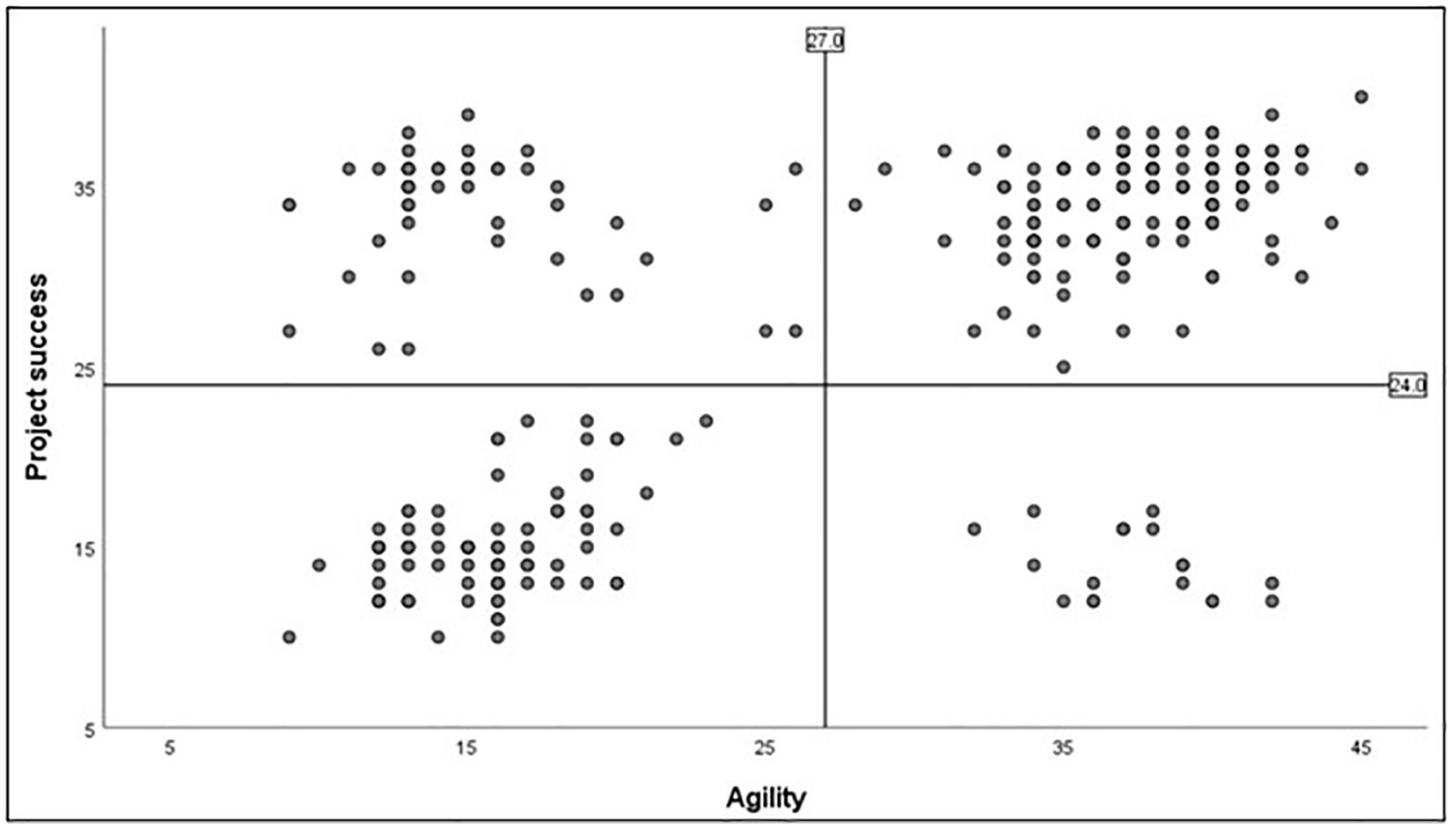
Scatter plot for agility and project success (cumulative). Source: own study. N = 286.

The scatter plot (Fig 2) illustrates survey participants’ cumulative responses concerning agility on horizontal axis < 9; 45 > and project success on vertical axis < 8; 40 >.

The horizontal line dividing the two sets is plotted at the level of a score of 24 points. This value was a borderline distinguishing successful projects from those considered a failure. The cluster above that line represents project success, while the cluster below that line illustrates failure. The vertical line dividing the two sets is plotted at the level of a score of 27 points. For this value, it is assumed that it was impossible to determine whether an agile approach was used. The cluster on the left represents absence of agility (traditional approach), while the cluster on the right represents the agile approach. On the basis of this figure it can be concluded that raising the level of agility may substantially increase the chances for project success.

**Fig 3:**
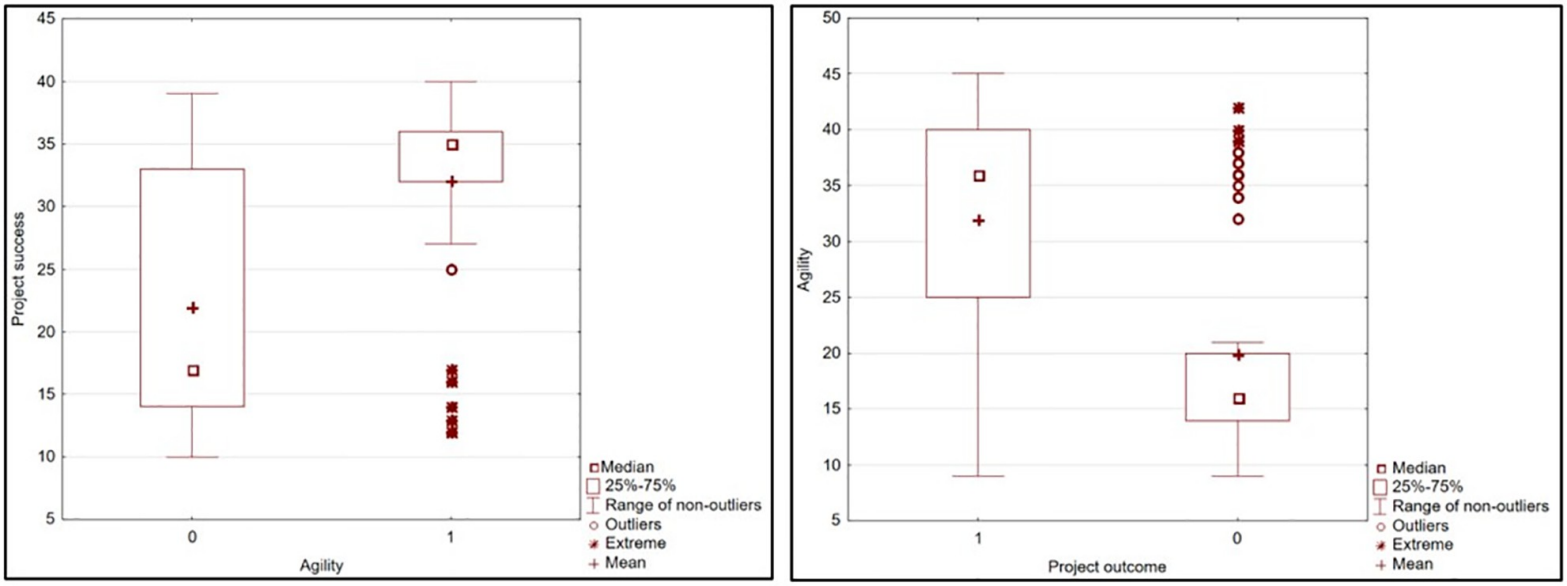
Project result and agile approach. Source: own study. N = 288.

Information on the project result where the respondents indicated the presence or absence of an agile approach provides more detail to the above analysis. (Fig 3). In both cases, the data on the vertical axis is presented as cumulative score, and on the horizontal axis using the total score converted into 0 (condition not met) and 1 (condition met).

Fig 3 clearly illustrates the existence of strong correlation between the approach applied and project success from two perspectives. It is demonstrated that: (1) a high level of agility increases the chance for project success; (2) successful projects are more frequent in organisations with higher level of agility. In conclusion, it has been demonstrated that the level of implementation of agile project management solutions in an enterprise influences project success potential (a correlation exists between these elements), thus **H4 can be positively verified**.

The study confirms that project success is more frequent where agile approach is applied (median 35 points, mean 32 points) than if this method is not implemented (median 17 points, mean 22 points). Extreme values were recorded where agile approach was present. These measurements were identified below the lower range of non-outliers, meaning that, despite the implementation of agile approach, a project turned out to be a failure (however, such cases were rare). From the opposite perspective, high level of agility (median 36 points, mean 32 points) was recorded for successful projects. For projects that failed, the median was only 16 points and the mean—20 points. Outliers and extreme values were recorded for project failure. These measurements were identified above the upper range of non-outliers, which means that, despite the high level of agility, a project turned out to be a failure (however, such cases were rare).

## Discussion of results

The aim of this study was to identify the factors determining project success from the perspective of defined elements of agile management. They were discussed in detail in the first part of the paper. Each factor was evaluated individually based on its importance during task performance. The further part of this paper is devoted to the correlation analysis of these factors and the components of project success both individually and in aggregated form.

In the adopted research model, the factor influencing project success is the application of agile approach in the implementation of project undertakings. First, using the identified agility indicators (9 elements) and project success components (8 elements), correlation analyses were performed. A total of 72 coefficients were obtained. The correlation results (Spearman’s ρ—rho) for agility components and project success components are presented in Table 7.

**Table 7:** Correlation results analysis (Spearman’s ρ)—Identified agility components / project success.

Agility components		Project success							
		Schedule	Budget	Functionality	Client satisfaction	Project team satisfaction	Benefits for product users	Benefits for the company	Strategic goals
People and interactions prevailing over tools and processes	Correlation coefficient	0.682**	0.583**	0.604**	0.508**	0.647**	0.549**	0.647**	0.601**
Working software prevailing over detailed documentation	Correlation coefficient	0.645**	0.612**	0.554**	0.677**	0.587**	0.588**	0.586**	0.600**
Client collaboration prevailing over contract negotiation	Correlation coefficient	0.622**	0.655**	0.638**	0.585**	0.598**	0.610**	0.562**	0.548**
Responding to changes in the course of work prevailing over following a plan	Correlation coefficient	0.570**	0.656**	0.423**	0.613**	0.621**	0.596**	0.603**	0.638**
Delivering project deliverables in an iterative, incremental manner	Correlation coefficient	0.651**	0.632**	0.525**	0.554**	0.598**	0.619**	0.623**	0.607**
The best architecture, requirements and design solutions originating from self-organising teams	Correlation coefficient	0.605**	0.628**	0.499**	0.615**	0.636**	0.614**	0.627**	0.632**
Maintaining good relationships with project stakeholders, characterised by mutual trust and cooperation	Correlation coefficient	0.707**	0.675**	0.627**	0.603**	0.742**	0.691**	0.661**	0.647**
Performance and functional criteria are used when evaluating tenders	Correlation coefficient	0.623**	0.624**	0.579**	0.599**	0.657**	0.660**	0.483**	0.610**
Project meetings (sprints) are organised frequently enough	Correlation coefficient	0.694**	0.634**	0.349**	0.613**	0.616**	0.607**	0.641**	0.633**

Source: own study. For all coefficients—significance (two-sided) p < 0,001. N = 288.

The strongest correlations were recorded for maintaining good relationships with project stakeholders and mutual trust and cooperation and keeping up with the schedule (0,707) and satisfaction of the project team (0,742). The smallest impact was recorded for project functionality—a moderate correlation in three cases was recorded [responding to changes in the course of work prevailing over following the plan (0,423), the best architectural solutions, requirements and designs originating from self-organising teams (0,499), project meetings (sprints) organised frequently enough (0,349)].

The survey results show that agile components presenting strong correlations (> 0,5) with all project success components analysed include: people and interactions prevailing over tools and processes; collaboration with the client prevailing over contract negotiations; delivering project deliverables in an iterative/incremental way, and maintaining good relationships with project stakeholders, characterised by mutual trust and cooperation. These results are the consequence of enterprises operating in a knowledge-based economy.

In the following part of the study a generalisation was made and the correlation of the grouped agility components with individual project success elements was examined. The analysis of the results (Spearman’s rank correlation coefficients) showed a strong correlation of the grouped agility components with all elements of project success: schedule (0.684), budget (0,699), functionality (0,706), client satisfaction (0,721), project team satisfaction (0,754), benefits for product clients (0,743), benefits for the company (0,755), achievement of strategic objectives (0,750).

In another generalization, the correlation between grouped agility components and project success was examined. Table 8 shows, the frequency (%) of project success or failure depending on agility components implemented in the company.

**Table 8:** Contingency table of project outcome and company agility.

		Project result		TOTAL
		Failure	Success	
Company agility	Absent	26%	18%	44%
	Present	7%	49%	56%
TOTAL		33%	67%	100%

Source: own study. N = 288

Based on the survey results it can be concluded that nearly half of the respondents (49%) who indicated agile approach to project management in the enterprise also achieved project success. The analysis clearly confirms that implementing agile approach definitely increases the probability of final project success. To complete the analyses concerning the relationship between agility and project success, the strength of correlation between these two components (agility and success) was verified. All the results obtained are statistically significant. The value of the chi-square test (χ^2^ = 70,835; *df* = 1; *p* < 0,001) indicates that correlation exists between agility and project success. The ϕ measure (*λ*(*x*) = 73,385; *df* = 1; *p* < 0,001, *φ* = 0,606; *p* < 0,001), Cramer’s V statistic (*V Cramera* = 0,606; *p* < 0,001) and Spearman’s rank correlation (0,606) reflect a strong correlation between project outcome and company agility.

In conclusion, it has been proven that correlation exists between the components of project success and individual components of agility both partially (in different approaches) and in a grouped approach. **The positive verification of H5 hypothesis confirmed the existence of strong correlation between enterprise agility and successful task performance**.

The adopted research model generally presented enterprise agility as a factor shaping project success. Therefore, an attempt was made to identify those agility components (out of 9 main components) that are particularly correlated to success (independent variables). Such identification was performed using regression analysis with all agility components as a basis. For that purpose, two modelling approaches were used: multiple regression (backward elimination method) and logistic regression (backward elimination method: reliability quotient). To verify collinearity between variables *VIF* Variance Inflation Factors were calculated.

In the multiple regression model, the final outcome of the work—i.e. cumulative score of project success—was adopted as the dependent variable. The results demonstrate that in the created model out of nine examined components statistical significance of three was below 0,05 (*p* < 0,05). The results are shown in Table 9.

**Table 9:** Linear regression results.

Model	Non-standardised coefficients		Standardised coefficients	T	Significance	Collinearity statistics	
	B	Standard error	Beta			Tolerance	VIF
(Constant)	18,188	1,299		14,004	0,000		
*Linp*	1,327	0,582	0,199	2,281	0,023	0,333	3,003
*Dosd*	1,205	0,531	0,179	2,268	0,024	0,406	2,461
*Dpit*	1,433	0,530	0,208	2,703	0,007	0,428	2,338

Source: own study. N = 288.

The model included the following elements: people and interactions prevailing over tools and processes (*linp*), working software prevailing over detailed documentation (*dosd*) and delivery in an iterative and incremental way (*dpit*) as shown in formula 2. Collinearity test value (*VIF*) for the variables in the model indicates that the collinearity between the predictors (1 < *VIF* < 10) is negligible, so it can be assumed that the collinearity problem is at an acceptable level. For the purposes of analysis, the overall company agility indicator was added to the regression model and the calculations were performed again. In the final model, only the added indicator remained (*sta*ł*a* = 24,153, *B* = 11,310, *p* < 0,05, *VIF* = 1,000), which confirms the previous conclusion.

Formula 2. Project success model I

$$S_{p}=18,188+1,327linp+1,205dosd+1,433dpit$$ (2)

Of the above-mentioned elements, the strongest influence on project success (*S*_*p*_) was recorded for delivering project deliverables in an iterative and incremental manner (*B* = 1,433). People and interactions prevailing over tools and processes (*B* = 1,327) and working software prevailing over detailed documentation (*B* = 1,205) are also part of the model, but their contribution is noticeably smaller. A partial conclusion can be drawn from the above formula that an increase in the cumulative level of the above elements increases the cumulative level of project success.

Additionally, logistic regression analysis was performed, where both project success (dependent variable) and all independent variables were assigned either 0 or 1 value. In this model (*logit P* = −0,374 + 1,696*LINP* + 0,892*RASZ*) the following elements were identified: people and interactions prevailing over tools and processes (*LINP*) and the best architectural solutions originating from self-organising teams (*RASZ*)—Table 10. The detailed form of the model is presented in formula 3.

**Table 10:** Logistic regression results.

Variables	B	Standard error	Wald	Df	Significance	Exp(B)
*LINP*	1,696	0,390	18,920	1	0.000	5.449
*RASZ*	0.892	0.380	5.499	1	0.019	2.441
(Constant)	-0.374	0.191	3.817	1	0.051	0.688

Source: own study. N = 288.

Formula 3. Project success Model II

$$P\left(X\right)=\frac{1}{1+e^{-\left(-0,374+1,696LINP+0,892RASZ\right)}}$$ (3)

The above model indicates project success or failure with high accuracy (percentage of total correct classifications—77.4; see Table 11). Based only on the information on human interactions and self-organising teams it is possible to foresee the project outcome (success or failure) with more than 77 percent accuracy.

**Table 11:** Classification table for Model II.

Observed		Expected		
		Success (0–1)		Percentage of accurate forecast
		0	1	
Success (0–1)	0	65	24	73.0
	1	41	158	79.4
Total percentage				77,4

Source: own study. N = 288.

Based of further analyses, the calculation of project success indicator values depending on the different values of the independent variables is shown in Table 12. For the success value to be higher than the cut-off point 0,5 the following elements are required: people and mutual interactions prevailing over tools and processes and/or the best architectural solutions originating from self-organised teams (value 1).

**Table 12:** Project success values *P*(*X*) depending on *LINP* and *RASZ*.

No.	LINP	RASZ	P(X)
1	0	0	0.408
2	0	1	0.627
3	1	0	0.790
4	1	1	0.902

Source: own study.

Based on Model II, some **conclusions and recommendations** for project success can be drawn.

1. Project success is influenced by *people and mutual interactions prevailing over tools and processes*. The chance of project success for an organisation where the described attitudes are present is considerably higher than in an organisation where such attitudes were not found (*e*^1,696 (1−0)^ = 5,452).
2. Project success is influenced by *self-organising teams*. The chance of project success for an organisation where teams are self-organised is considerably higher than in an organisation where such behaviour was not found (*e*^0,892 (1−0)^ = 2,440).

In conclusion, the entire research material collected and the analyses conducted **unambiguously confirm the validity of H1**, which means that a proper configuration of agile components is a prerequisite for success in project implementation. The correlation analyses presented above clearly demonstrate the existence of interrelation between agility and project success. Its influence was recorded both for individual success components as well as success in cumulative terms. This means that agility is a necessary factor (prerequisite) for project success. The analyses demonstrate a correlation between project success and agility. An attempt was also made to create a model of that relationship. Organisations striving for project success should ensure high level of agile management components.

The survey conducted and the results obtained complement the research gap identified in the literature on the subject of project success from the perspective of the agile approach [80–83]. Research to date has mainly focused on demonstrating overall relationship between project success and agile approach, with lesser focus on identifying the correspondence between individual components of both success and agile approach. The literature review indicates the critical factors for the successful implementation of agile approach [84] and key determinants of success in project ventures [85], but the juxtaposition of these elements and the search for mutual correlations shall be a direction for further research. The identified relationship that people and mutual interactions prevailing over tools and processes and self-organising teams affect the success of implemented tasks constitute a practical guideline that can serve as a basis for developing innovative management strategies focused on maximizing the probability of project success [86, 87]. The application of logistic regression demonstrated that implementing one of the core principles of the Agile Manifesto (people and mutual interactions prevailing over tools and processes) contributes to a more than fivefold (*e*^1,696 (1−0)^ = 5,452) increase in the probability of project success, and one of the core principles of agile approach (self-organising teams) contributes to a more than twofold (*e*^0,892 (1−0)^ = 2,440) increase in the probability of achieving the set goals. Further research has shown that building self-organising teams alone conduces to more than a 60% (62.7%) probability of success, while project work founded on on people and their mutual interactions results in almost 80% (79%) certainty of successful task completion. In turn, application of both elements together contributes to over 90% (90.2%) certainty of success. The developed model is highly accurate (percentage of all correct classifications—77.4), therefore the results can be assumed to be reliable. The authors would also like to point out that in addition to the research presented in the text, they conducted a number of other analyses including e.g. analysis of moderation, mediation, verification of the possible collider bias effect. These analyses were carried out to eliminate the potential mutual influence of different variables. As no disturbing interdependencies or influences were identified, the paper only includes the results of the key studies to achieve the research objective.

The contribution of the presented research to the economy seems to be significant. In Poland, the value of ICT industry grew by as much as 19% in 2021. In contrast, the reported growth of the same value between 2019 and 2020 was only 9%. The ICT industry has shown once again its strength and resilience, as well as its products and services proved to be indispensable in difficult times, related to the pandemic, the energy crisis and general geopolitical uncertainty. More and more companies are becoming aware that without proper IT tools, not only will they not gain a competitive advantage, but they could simply disappear from the market [87]. For the above reasons, the need to strive for project success becomes imperative. The research results presented herein can serve as practical guideline for achieving the set project goals also in companies operating in other branches of industry.

## Limitations and future research

The described study provides a better understanding of the relationship between project success and various elements of agile approach. It should be explicitly emphasised that the study was limited to the ICT industry. Despite the multifaceted analysis of the subject matter, further research needs to be continued, especially in other industries. A particularly interesting aspect of the study seems to be the detailed analysis of individual cases (case studies), mainly projects of high complexity and very high budget. The study also emphasised relatively poor relations between the academics and entrepreneurs. This phenomenon requires further analysis, and an attempt to reduce this gap should be a in the spotlight both for the academics and the business community.

The limitations of the described study include the arbitrary method of converting the data collected in survey questionnaires to 0–1 values (binary representation of presence or absence of a factor). For the purpose of the analyses, a delimitation at 3n was adopted (min. 1n; max. 5n). Determining the exact point of transition between 0 and 1 could be the subject of further research. Further research could also address the elements that were evaluated the lowest by the respondents or seek to obtain more accurate regression models.

At this point it should be noted that each of the variables could exert potentially some influence on another. For example, the period of company’s operation could affect project agility (companies with a longer history may manage projects in a less agile way), agility can influence the project team satisfaction or vice versa: a satisfied team could be more willing to work in an agile manner. In addition, the study relates to *post factum* project observations, therefore in some cases it was possible that in the course of the project managers changed their management style taking into account their subjective assessment of the probability of project success (their management style could have become more rigid when project failure was anticipated). These limitations to the study should be noted. The test values *VIF* demonstrate slight collinearity between the predictors, hence it may be concluded that the correlations observed and the relationships shown in the models still provide a useful picture of the impact of agile management on project success.

## Conclusions

The subject matter and the research objective was to identify various components of agility as project success factors. The factors were evaluated on the basis of their impact and significance for actual project implementation according to eight criteria. The factors were evaluated by survey respondents in terms of their impact and frequency. Survey results were calculated using statistical methods such as cross-tabulation, correlation analysis, regression analysis, and statistical tests. An author’s formula for determining the values of indicators (*W*) of meeting the set criteria was also developed.

The analysis of survey results allowed the main study objective to be achieved. It concerns in particular the identification of relations between selected agility components and project success components. Identification of such relationships allows recommendations to be formulated for solutions and attitudes increasing the chances for success in project management. It should be noted that agility proved to be strongly correlated with all components of project success. Therefore, it can be concluded that the presence and high level of agility is conducive to project success. Moreover, it is worth noting that project success is achieved to a greater extent where an agile approach is used than where it is used to a lesser extent. Multidimensional analysis of project success has led to a final conclusion that the use of advanced project management solutions is a necessary but not a sufficient condition for project success To succeed, additional elements supporting project activities are required. This concerns two of them in particular, i.e. people and interactions prevailing over tools and processes and the best architectural solutions originating from self-organised teams which are crucial for positive evaluation of project activities. These two elements considerably increase the probability of project success. The answers provided to individual questions, as well as the positive verification of the assumptions made and the overall analysis of the collected research material unequivocally confirm the correctness of the above conclusion. It should be emphasised, however, that fulfilling only one agile condition does not ensure project success. It is the synergy of individual agile components applied that increases the chances for project success.

## Supporting information

S1 Fig Research model scheme. Own study.
(TIF)

S2 Fig Scatter plot for agility and project success (cumulative). Own study. N = 286.
(TIF)

S3 Fig Project result and agile approach. Own study. N = 288.
(TIF)

S1 Table Characteristics of the survey sample. Own study. N = 288.
(DOCX)

S2 Table Identification of the extent of involvement of individual agility components in project task implementation. Own study. N = 288.
(DOCX)

S3 Table Assessment of agility components (%). Own study. N = 288.
(DOCX)

S4 Table Test results for individual components of project success. Own study. N = 288.
(DOCX)

S5 Table Ratings of project success components (%). Own study. N = 288.
(DOCX)

S6 Table Impact of the adopted project management method on project result. Own study. N = 288.
(DOCX)

S7 Table Correlation results analysis (Spearman’s ρ)—Identified agility components / project success. Own study. For all coefficients—significance (two-sided)p<0,001. N = 288.
(DOCX)

S8 Table Contingency table of project outcome and company agility. Own study. N = 288.
(DOCX)

S9 Table Linear regression results. Own study. N = 288.
(DOCX)

S10 Table Logistic regression results. Own study. N = 288.
(DOCX)

S11 Table Classification table for Model II. Own study. N = 288.
(DOCX)

S12 Table Project success values P(X) depending on LINP and RASZ. Own study.
(DOCX)

S1 Formula General formula for the determination of the indicator (W). (DOCX)

S2 Formula Project success model I. (DOCX)

S3 Formula Project success Model II. (DOCX)
