# Supplementary figures and images for: The impact of identified agility components on project success—ICT industry perspective
Source: PLoS One. 2023 Mar 23;18(3):e0281936. doi: 10.1371/journal.pone.0281936 (PMC10035824; doi:10.1371/journal.pone.0281936)

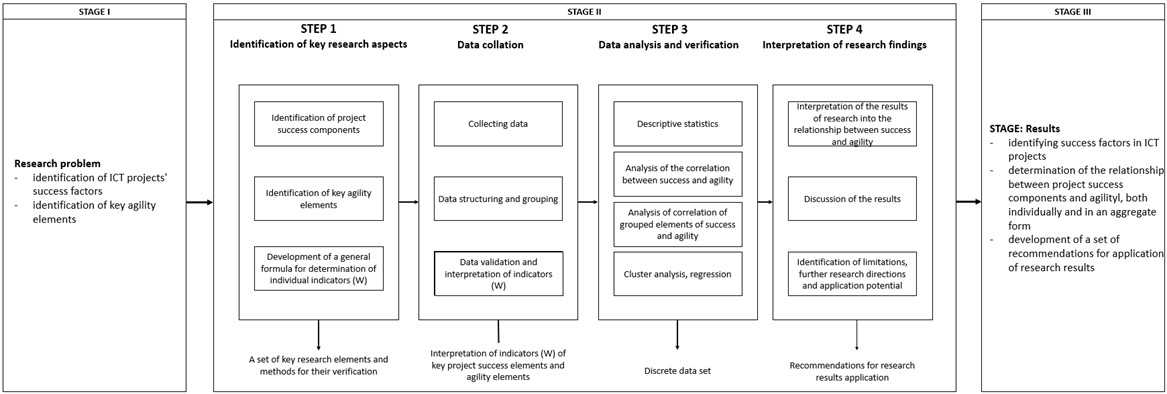

Supplement: S1 Fig — Own study. (TIF) [file pone.0281936.s001.tif]

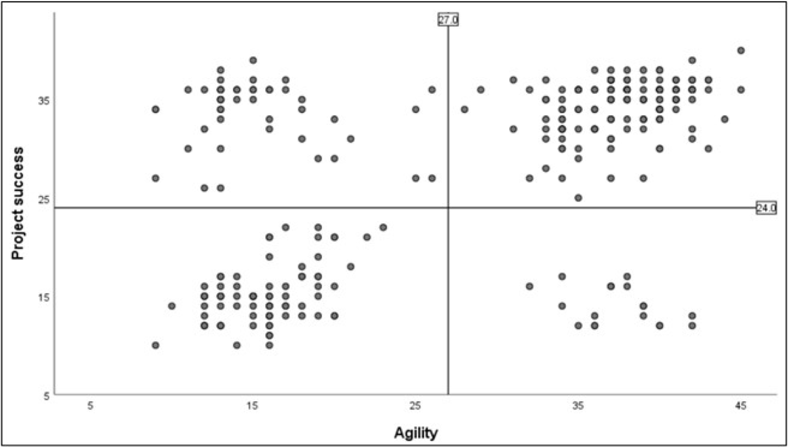

Supplement: S2 Fig — Own study. N = 286. (TIF) [file pone.0281936.s002.tif]

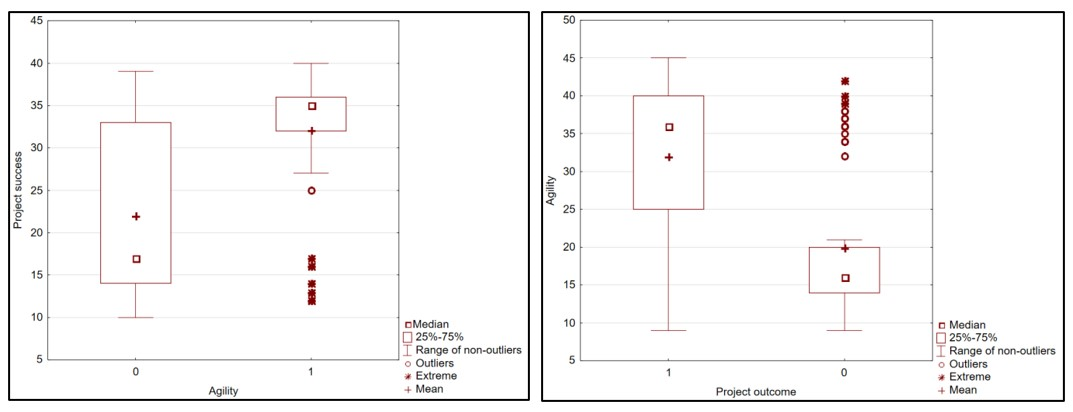

Supplement: S3 Fig — Own study. N = 288. (TIF) [file pone.0281936.s003.tif]
