# Supplementary material for: The impact of identified agility components on project success—ICT industry perspective
Source: PLoS One. 2023 Mar 23;18(3):e0281936. doi: 10.1371/journal.pone.0281936 (PMC10035824; doi:10.1371/journal.pone.0281936)
Supplement: S1 Table — Own study. N = 288. (DOCX) [file pone.0281936.s004.docx]

**Table1. Characteristics of the survey sample**

| **Characteristics of enterprises represented by survey respondents** | |
| --- | --- |
| Role of enterprises in project implementation | |
| Contractor | 85% |
| Employer | 15% |
| Categories of surveyed enterprises by size | |
| Small | 17% |
| Medium | 70% |
| Large | 13% |
| Period of operation of the company on the market | |
| Up to 1 year | 2% |
| Between 1 and 5 years | 14% |
| Between 6 and 10 years | 62% |
| Over 10 years | 22% |
| Discipline | |
| IT | 54% |
| Telecommunications | 23% |
| Energy | 10.7% |
| Industry | 4% |
| Financial services | 2% |
| Marketing and advertising | 2% |
| Construction | 0.3% |
| Other (medical, military) | 4% |
| **Characteristics of respondents participating in the survey** | |
| Age of respondents | |
| Up to 25 years | 4% |
| Between 26 and 35 years | 24% |
| Between 36 and 50 years | 68% |
| Over 50 years | 4% |
| Characteristics of respondents' jobs | |
| Product user | 4% |
| Project team member | 12% |
| Project manager | 31% |
| Lower/middle level manager | 18% |
| High-level manager | 28% |
| President/Member of the Board | 7% |
| Respondents' professional experience | |
| Less than 1 year | 6% |
| Between 2 and 5 years | 24% |
| Between 6 and 10 years | 49% |
| Over 10 years | 21% |
| Territorial area of the implemented projects | |
| Nation-wide projects | 30% |
| International projects | 70% |
| Number of people involved in the implementation of a single project | |
| From 1 to 10 persons | 19% |
| From 11 to 50 persons | 55% |
| From 51 to 100 persons | 22% |
| More than 100 people | 4% |
| Complexity of the projects | |
| Simple projects | 4% |
| Moderately complex projects | 21% |
| Complex projects | 62% |
| Highly complex projects | 13% |

*Source: own study. N=288.*
