# Supplementary material for: The impact of identified agility components on project success—ICT industry perspective
Source: PLoS One. 2023 Mar 23;18(3):e0281936. doi: 10.1371/journal.pone.0281936 (PMC10035824; doi:10.1371/journal.pone.0281936)
Supplement: S2 Table — Own study. N = 288. (DOCX) [file pone.0281936.s005.docx]

**Table 2. Identification of the extent of involvement of individual agility components in project task implementation**

| **Agility components** | **Mean**  $\bar{\boldsymbol{X}}$ | **Median**  $\boldsymbol{M(X)}$ | **Mode**  $\boldsymbol{D(}\boldsymbol{X}\boldsymbol{)}$ | **Standard deviation**  $\boldsymbol{S(}\boldsymbol{X}\boldsymbol{)}$ | **Skewness**  $\boldsymbol{A(}\boldsymbol{X}\boldsymbol{)}$ | **Kurtosis**  $\boldsymbol{K(}\boldsymbol{X}\boldsymbol{)}$ |
| --- | --- | --- | --- | --- | --- | --- |
| People and interactions prevailing over tools and processes | 3,14 | 4,00 | 4,00 | 1,575 | -0,188 | -1,604 |
| Working software prevailing over detailed documentation | 3,20 | 4,00 | 4,00 | 1,561 | -0,238 | -1,569 |
| Client collaboration prevailing over contract negotiation | 2,88 | 4,00 | 4,00 | 1,549 | -0,018 | -1,635 |
| Responding to changes in the course of work prevailing over following a plan | 3,30 | 2,00 | 5,00 | 1,535 | -0,267 | -1,543 |
| Delivering project deliverables in an iterative, incremental manner | 3,07 | 4,00 | 4,00 | 1,525 | -0,213 | -1,564 |
| The best architecture, requirements and design solutions originating from self-organising teams | 3,27 | 4,00 | 5,00 | 1,590 | -0,232 | -1,614 |
| Maintaining good relationships with project stakeholders, characterised by mutual trust and cooperation | 3,07 | 4,00 | 4,00 | 1,539 | -0,187 | -1,579 |
| Performance and functional criteria used when evaluating offers (samples, system demonstrations) | 3,20 | 4,00 | 4,00 | 1,531 | -0,190 | -1,566 |
| Project meetings (sprints) organised frequently enough | 3,07 | 4,00 | 4,00 | 1,525 | 0,189 | -1,566 |

Source: own study. N=288.
