# Supplementary material for: The impact of identified agility components on project success—ICT industry perspective
Source: PLoS One. 2023 Mar 23;18(3):e0281936. doi: 10.1371/journal.pone.0281936 (PMC10035824; doi:10.1371/journal.pone.0281936)
Supplement: S3 Table — Own study. N = 288. (DOCX) [file pone.0281936.s006.docx]

**Table 3. Assessment of agility components (%)**

| **Project success components** | **Negative verification of the indicator** | **Inconclusive** | **Positive verification of the indicator** |
| --- | --- | --- | --- |
| People and interactions prevailing over tools and processes | 44% | 0% | 56% |
| Working software prevailing over detailed documentation | 43% | 0% | 57% |
| Client collaboration prevailing over contract negotiation | 51% | 0% | 49% |
| Responding to changes in the course of work prevailing over following a plan | 42% | 0% | 58% |
| Delivering project deliverables in an iterative, incremental manner | 43% | 0% | 57% |
| The best architecture, requirements and design solutions originating from self-organising teams | 43% | 0% | 57% |
| Maintaining good relationships with project stakeholders, characterised by mutual trust and cooperation | 44% | 0% | 56% |
| Performance and functional criteria used when evaluating offers | 44% | 0% | 56% |
| Project meetings (sprints) organised frequently enough | 42% | 0% | 58% |

*Source: own study. N=288.*
