# Supplementary material for: The impact of identified agility components on project success—ICT industry perspective
Source: PLoS One. 2023 Mar 23;18(3):e0281936. doi: 10.1371/journal.pone.0281936 (PMC10035824; doi:10.1371/journal.pone.0281936)
Supplement: S4 Table — Own study. N = 288. (DOCX) [file pone.0281936.s007.docx]

**Table 4. Test results for individual components of project success**

| **Project success components** | **Mean**  $\bar{\boldsymbol{X}}$ | **Median**  $\boldsymbol{M(X}$**)** | **Mode**  $\boldsymbol{D(}\boldsymbol{X}\boldsymbol{)}$ | **Standard deviation**  $\boldsymbol{S(}\boldsymbol{X}\boldsymbol{)}$ | **Skewness**  $\boldsymbol{A(}\boldsymbol{X}\boldsymbol{)}$ | **Kurtosis**  $\boldsymbol{K(}\boldsymbol{X}\boldsymbol{)}$ |
| --- | --- | --- | --- | --- | --- | --- |
| Keeping up with budget | 3,56 | 4,00 | 4,00 | 1,413 | -0,647 | -1,047 |
| Keeping up with schedule | 3,43 | 4,00 | 4,00 | 1,398 | -0,553 | -1,143 |
| Ensuring functionality | 3,32 | 4,00 | 4,00 | 1,400 | -0,493 | -1,224 |
| Client's satisfaction | 3,58 | 4,00 | 5,00 | 1,489 | -0,669 | -1,107 |
| Satisfaction of project teams’ members | 3,35 | 4,00 | 4,00 | 1,484 | -0,477 | -1,313 |
| Ensuring benefits for the recipients of the project products | 3,50 | 4,00 | 4,00 | 1,374 | -0,628 | -1,021 |
| Ensuring technical, organisational, social, political and business benefits | 3,53 | 4,00 | 4,00 | 1,448 | -0,656 | -1,068 |
| Achieving the strategic objectives of the company | 3,46 | 4,00 | 5,00 | 1,502 | -0,416 | -1,427 |

*Source: own study. N=288.*
