# Supplementary material for: The impact of identified agility components on project success—ICT industry perspective
Source: PLoS One. 2023 Mar 23;18(3):e0281936. doi: 10.1371/journal.pone.0281936 (PMC10035824; doi:10.1371/journal.pone.0281936)
Supplement: S5 Table — Own study. N = 288. (DOCX) [file pone.0281936.s008.docx]

**Table 5. Ratings of project success components (%)**

| **Project success components** | **Negative verification of the indicator** | **Inconclusive** | **Positive verification of the indicator** |
| --- | --- | --- | --- |
| Keeping up with budget | 32% | 0% | 68% |
| Keeping up with schedule | 34% | 0% | 66% |
| Ensuring functionality | 36% | 0% | 64% |
| Client's satisfaction | 32% | 0% | 68% |
| Satisfaction of project teams’ members | 36% | 0.5% | 63.5% |
| Ensuring benefits for the recipients of the project outputs | 32% | 0% | 68% |
| Ensuring technical, organisational, social, political and business benefits | 35% | 0% | 65% |
| Achieving the strategic objectives of the company | 32% | 0% | 68% |

*Source: own study. N=288.*
