# Supplementary material for: The impact of identified agility components on project success—ICT industry perspective
Source: PLoS One. 2023 Mar 23;18(3):e0281936. doi: 10.1371/journal.pone.0281936 (PMC10035824; doi:10.1371/journal.pone.0281936)
Supplement: S6 Table — Own study. N = 288. (DOCX) [file pone.0281936.s009.docx]

**Table 6. Impact of the adopted project management method on project result**

|  | | **Project result** | | **TOTAL** |
| --- | --- | --- | --- | --- |
|  |  | **Failure** | **Success** |  |
| Project management method | None | 6% | 4% | 10% |
|  | Standard | 18% | 15% | 33% |
|  | Agile | 8% | 48% | 57% |
| SUM | | 33% | 67% | 100% |

Source: own study. N=288.
