# Supplementary material for: The impact of identified agility components on project success—ICT industry perspective
Source: PLoS One. 2023 Mar 23;18(3):e0281936. doi: 10.1371/journal.pone.0281936 (PMC10035824; doi:10.1371/journal.pone.0281936)
Supplement: S7 Table — Own study. For all coefficients—significance (two-sided)p<0,001. N = 288. (DOCX) [file pone.0281936.s010.docx]

**Table 7. Correlation results analysis (Spearman's ρ) - Identified agility components / project success**

| **Agility components** | | **Project success** | | | | | | | |
| --- | --- | --- | --- | --- | --- | --- | --- | --- | --- |
|  |  | **Schedule** | **Budget** | **Functionality** | **Client satisfaction** | **Project team satisfaction** | **Benefits for product users** | **Benefits for the company** | **Strategic goals** |
| People and interactions prevailing over tools and processes | Correlation coefficient | 0.682** | 0.583** | 0.604** | 0.508** | 0.647** | 0.549** | 0.647** | 0.601** |
| Working software prevailing over detailed documentation | Correlation coefficient | 0.645** | 0.612** | 0.554** | 0.677** | 0.587** | 0.588** | 0.586** | 0.600** |
| Client collaboration prevailing over contract negotiation | Correlation coefficient | 0.622** | 0.655** | 0.638** | 0.585** | 0.598** | 0.610** | 0.562** | 0.548** |
| Responding to changes in the course of work prevailing over following a plan | Correlation coefficient | 0.570** | 0.656** | 0.423** | 0.613** | 0.621** | 0.596** | 0.603** | 0.638** |
| Delivering project deliverables in an iterative, incremental manner | Correlation coefficient | 0.651** | 0.632** | 0.525** | 0.554** | 0.598** | 0.619** | 0.623** | 0.607** |
| The best architecture, requirements and design solutions originating from self-organising teams | Correlation coefficient | 0.605** | 0.628** | 0.499** | 0.615** | 0.636** | 0.614** | 0.627** | 0.632** |
| Maintaining good relationships with project stakeholders, characterised by mutual trust and cooperation | Correlation coefficient | 0.707** | 0.675** | 0.627** | 0.603** | 0.742** | 0.691** | 0.661** | 0.647** |
| Performance and functional criteria are used when evaluating tenders | Correlation coefficient | 0.623** | 0.624** | 0.579** | 0.599** | 0.657** | 0.660** | 0.483** | 0.610** |
| Project meetings (sprints) are organised frequently enough | Correlation coefficient | 0.694** | 0.634** | 0.349** | 0.613** | 0.616** | 0.607** | 0.641** | 0.633** |

Source: own study. For all coefficients - significance (two-sided)$p<0,001$. N=288.
