# Supplementary material for: The impact of identified agility components on project success—ICT industry perspective
Source: PLoS One. 2023 Mar 23;18(3):e0281936. doi: 10.1371/journal.pone.0281936 (PMC10035824; doi:10.1371/journal.pone.0281936)
Supplement: S8 Table — Own study. N = 288. (DOCX) [file pone.0281936.s011.docx]

**Table 8. Contingency table of project outcome and company agility**

|  |  |  | **Project result** | | **TOTAL** |
| --- | --- | --- | --- | --- | --- |
|  |  |  | **Failure** | **Success** |  |
| Company agility | | Absent | 26% | 18% | 44% |
|  |  | Present | 7% | 49% | 56% |
| TOTAL | | | 33% | 67% | 100% |

Source: own study. N=288.
