# Supplementary material for: The impact of identified agility components on project success—ICT industry perspective
Source: PLoS One. 2023 Mar 23;18(3):e0281936. doi: 10.1371/journal.pone.0281936 (PMC10035824; doi:10.1371/journal.pone.0281936)
Supplement: S9 Table — Own study. N = 288. (DOCX) [file pone.0281936.s012.docx]

**Table 9. Linear regression results**

| **Model** | **Non-standardised coefficients** | | **Standardised coefficients** | **T** | **Significance** | **Collinearity statistics** | |
| --- | --- | --- | --- | --- | --- | --- | --- |
|  | **B** | **Standard error** | **Beta** |  |  | **Tolerance** | **VIF** |
| (Constant) | 18,188 | 1,299 |  | 14,004 | 0,000 |  |  |
| *Linp* | 1,327 | 0,582 | 0,199 | 2,281 | 0,023 | 0,333 | 3,003 |
| *Dosd* | 1,205 | 0,531 | 0,179 | 2,268 | 0,024 | 0,406 | 2,461 |
| *Dpit* | 1,433 | 0,530 | 0,208 | 2,703 | 0,007 | 0,428 | 2,338 |

*Source: own study. N=288.*
