# Supplementary material for: The impact of identified agility components on project success—ICT industry perspective
Source: PLoS One. 2023 Mar 23;18(3):e0281936. doi: 10.1371/journal.pone.0281936 (PMC10035824; doi:10.1371/journal.pone.0281936)
Supplement: S10 Table — Own study. N = 288. (DOCX) [file pone.0281936.s013.docx]

**Table 10. Logistic regression results**

| **Variables** | **B** | **Standard error** | **Wald** | **Df** | **Significance** | **Exp(B)** |
| --- | --- | --- | --- | --- | --- | --- |
| *LINP* | 1,696 | 0,390 | 18,920 | 1 | 0.000 | 5.449 |
| *RASZ* | 0.892 | 0.380 | 5.499 | 1 | 0.019 | 2.441 |
| (Constant) | -0.374 | 0.191 | 3.817 | 1 | 0.051 | 0.688 |

*Source: own study. N=288.*
