# Supplementary material for: The impact of identified agility components on project success—ICT industry perspective
Source: PLoS One. 2023 Mar 23;18(3):e0281936. doi: 10.1371/journal.pone.0281936 (PMC10035824; doi:10.1371/journal.pone.0281936)
Supplement: S11 Table — Own study. N = 288. (DOCX) [file pone.0281936.s014.docx]

**Table 11. Classification table for model II**

| **Observed** | | **Expected** | | |
| --- | --- | --- | --- | --- |
|  |  | **Success (0-1)** | | **Percentage of accurate forecast** |
|  |  | **0** | **1** |  |
| Success (0-1) | 0 | 65 | 24 | 73.0 |
|  | 1 | 41 | 158 | 79.4 |
| Total percentage | |  |  | 77,4 |

*Source: own study. N=288.*
