# Supplementary material for: The impact of identified agility components on project success—ICT industry perspective
Source: PLoS One. 2023 Mar 23;18(3):e0281936. doi: 10.1371/journal.pone.0281936 (PMC10035824; doi:10.1371/journal.pone.0281936)
Supplement: S12 Table — Own study. (DOCX) [file pone.0281936.s015.docx]

**Table 12. Project success values** $\boldsymbol{P(X)}$ **depending on** $\boldsymbol{LINP}$ **and** $\boldsymbol{RASZ}$

| **No.** | **LINP** | **RASZ** | **P(X)** |
| --- | --- | --- | --- |
| 1 | 0 | 0 | 0.408 |
| 2 | 0 | 1 | 0.627 |
| 3 | 1 | 0 | 0.790 |
| 4 | 1 | 1 | 0.902 |

*Source: own study.*
