# Supplementary material for: The impact of identified agility components on project success—ICT industry perspective
Source: PLoS One. 2023 Mar 23;18(3):e0281936. doi: 10.1371/journal.pone.0281936 (PMC10035824; doi:10.1371/journal.pone.0281936)
Supplement: S1 Formula — (DOCX) [file pone.0281936.s016.docx]

Formula 1. General formula for the determination of the indicator $\left( W \right)$

$W=\left\{ \begin{aligned} 0, for \sum_{i=1}^{n} x_{i}<3n \\ unconclusive, for \sum_{i=1}^{n} x_{i}=3n \\ 1, for \sum_{i=1}^{n} x_{i}>3n \end{aligned} \right.$ (1)

$W$ - designated indicator,

$n$ - number of elements (taken into account in creating the indicator),

$i$ - consecutive number of the analysed response (assessment),

$x_{i}$- value of the response (assessment), expressed by the respondent for the n^th^ object included in the indicator, $x_{i}\in\left\{ 1,2,3,4,5 \right\}$.
