# Supplementary material for: The impact of identified agility components on project success—ICT industry perspective
Source: PLoS One. 2023 Mar 23;18(3):e0281936. doi: 10.1371/journal.pone.0281936 (PMC10035824; doi:10.1371/journal.pone.0281936)
Supplement: S2 Formula — (DOCX) [file pone.0281936.s017.docx]

Formula 2. Project success model I

$S_{p}=18,188+1,327linp+1,205dosd+1,433dpit$ (2)
