# Supplementary material for: The impact of identified agility components on project success—ICT industry perspective
Source: PLoS One. 2023 Mar 23;18(3):e0281936. doi: 10.1371/journal.pone.0281936 (PMC10035824; doi:10.1371/journal.pone.0281936)
Supplement: S3 Formula — (DOCX) [file pone.0281936.s018.docx]

Formula 3. Project success Model II

$P\left( X \right)=\frac{1}{1+e^{-(-0,374+1,696LINP+0,892RASZ)}}$ (3)
